# On the relationship between citations of publication output and Hirsch index *h* of authors: conceptualization of tapered Hirsch index *h*_T_, circular citation area radius *R* and citation acceleration *a*

**DOI:** 10.1007/s11192-012-0805-7

**Published:** 2012-07-10

**Authors:** Keshra Sangwal

**Affiliations:** Department of Applied Physics, Lublin University of Technology, ul. Nadbystrzycka 38, 20-618 Lublin, Poland

**Keywords:** Citations *L*, Citation acceleration *a*, Circular citation area radius *R*, Ferrers diagrams, Hirsch constant *A* = *L*/*h*^2^, Hirsch index *h*, Publication output, Tapered *h* index (*h*_T_)

## Abstract

The nature of the empirical proportionality constant *A* in the relation *L* = *Ah* ^2^ between total number of citations *L* of the publication output of an author and his/her Hirsch index *h* is analyzed using data of the publication output and citations for six scientists elected to the membership of the Royal Society in 2006 and 199 professors working in different institutions in Poland. The main problem with the *h* index of different authors calculated by using the above relation is that it underestimates the ranking of scientists publishing papers receiving very high citations and results in high values of *A*. It was found that the value of the Hirsch constant *A* for different scientists is associated with the discreteness of *h* and is related to the tapered Hirsch index *h*_T_ by *A* ^1/2^ ≈ 1.21*h*_T_. To overcome the drawback of a wide range of *A* associated with the discreteness of *h* for different authors, a simple index, the radius *R* of circular citation area, defined as *R* = (*L*/π)^1/2^ ≈ *h*, is suggested. This circular citation area radius *R* is easy to calculate and improves the ranking of scientists publishing high-impact papers. Finally, after introducing the concept of citation acceleration *a* = *L*/*t* ^2^ = π(*R*/*t*)^2^ (*t* is publication duration of a scientist), some general features of citations of publication output of Polish professors are described in terms of their citability. Analysis of the data of Polish professors in terms of citation acceleration *a* shows that: (1) the citability of the papers of a majority of physics and chemistry professors is much higher than that of technical sciences professors, and (2) increasing fraction of conference papers as well as non-English papers and engagement in administrative functions of professors result in decreasing citability of their overall publication output.

**Electronic supplementary material:**

The online version of this article (doi:10.1007/s11192-012-0805-7) contains supplementary material, which is available to authorized users.

## Introduction

For over two decades there has been an increasing interest in the evaluation of the scientific research output of scientists in terms of numerical indexes quantifying it unequivocally. During the last 6 years, the introduction of *h* index by Hirsch (2005) has provided enormous impetus in finding tools to quantify the research output of individual scientists, university faculties and research institutions. The Hirsch index *h* is defined as the highest number of papers of an author that received *h* or more citations. The main drawback of the *h* index of an author is that it does not count citations received by *h* papers with citations *l*_*n*_ > *h* and (*n* − *h*) papers having citations *l*_*n*_ < *h.* Here *l*_*n*_ denotes the number of citations of an individual paper of rank *n* such that the total number of citations *L* is the sum of citations coming from individual papers. In order to improve and modify the *h* index several contributions have been devoted (for example, see: Alonso et al. 2009; Burrell 2009; Egghe 2010; Franceschini and Maisano 2010a, b; Glänzel and Schubert 2010; Jin et al. 2007; Kosmulski 2006; Navon 2009). Among the different *h* variants, the tapered *h* index (*h*_T_), proposed by Anderson et al. (2008), is one which takes into account the total number of citations.

In his classic work Hirsch (2005) found that there is a relationship between the total number *L* of citations and the Hirsch index *h*, given by

1 $$ L = Ah^{2} , $$

where the empirical proportionality constant *A*, called Hirsch constant hereafter, lies in a wide range. The main problem with the *h* index is that it underestimates the ranking of scientists publishing papers receiving very high citations and results in high values of *A*.

The aim of the present paper is fourfold: (1) to analyze the nature of the Hirsch constant *A* of the publication output of different professors working in different institutions in Poland and their Hirsch index *h*, (2) to propose a simple non-integer index, the radius *R* of circular citation area, defined as *R* = (*L*/π)^1/2^ ≈ *h*, which is easy to calculate and improves the ranking of scientists publishing high-impact papers, (3) to describe some general features of citations of publication output of Polish scientists in terms of their citability from the concept of citation acceleration *a* = *L*/*t* ^2^ = π(*R*/*t*)^2^ (*t* is publication duration of a scientist), and (4) to discuss the influence of conference papers, non-English papers and administrative functions of professors on the citability of their overall publication output.

## Selection of authors and their citation data for analysis

For the analysis we considered the publication output and citations for six scientists elected to the membership of the Royal Society in 2006 and 199 professors (i.e., academic staff holding habilitation degrees and national titles of professors) working in different institutions in Poland. These different institutions are listed in Table 1. Among the selected Polish professors, 80 are specialized in physical sciences (Ph), 53 are specialized in chemical sciences (Ch) whereas 66 are specialized in technical sciences (T). All relevant data are given in Appendixes A1–A6 of Online Supplement).

**Table 1:** Polish Institutions considered in analysis

Institution*	Abbreviations	Disciplines	Professors analyzed
University of Warsaw (Uniwersytet Warszawski)	UW	Ph, Ch	14
University of Wrocław (Uniwersytet Wrocławski)	UWroc	Ph, Ch	14
University of Silesia, Katowice (Uniwersytet Śląski, Katowice)	US	Ph, Ch,T	13
Maria Curie-Skłodowska Univerisity (Uniwersytet Marii Curie-Skłodowska, Lublin)	UMCS	Ph, Ch	11
University of Opole (Uniwersytet Opolski)	UO	Ph, Ch	5
University of Kazimierz Wielki, Bydgoszczy (Uniwersytet im. Kazimierza Wielkiego, Bydgoszcz)	UKW	Ph, Ch	2
Warsaw University of Technology (Politechnika Warszawska)	WUT	Ph, Ch, T	17
Łódź University of Technology (Politechnika Łódzka)	LoUT	Ph, Ch,T	18
Gdańsk University of Technology (Politechnika Gdańska)	GUT	Ph, Ch, T	20
Military University of Technology, Warsaw (WAT im. Jarosława Dąbrowskiego, Warsaw)	WAT	Ph, Ch, T	10
Czestochowa University of Technology (Politechnika Częstochowska)	CzUT	Ph, Ch, T	15
UTP of J.J. Sniadeckis, Bydgoszczy (UTP im. J. i J. Śniadeckich w Bydgoszczy)	UTP	Ph, Ch, T	5
Koszalin University of Technology (Politechnika Koszalińska)	KUT	Ph, Ch, T	8
Technical-Humanities Academy, Bielsko-Biała (Akademia Techniczno-Humanistyczna, Bielsko-Biała)	ATH	Ph, Ch, T	4
Institute of Low Temperature and Structural Research PASc (Instytut Niskich Temperatur I Badań Strukturalnych PAN), Wrocław	ILTSR	Ph	15
Institute of Physical Chemistry PASc (Instytut Chemii Fizycznej PAN), Warsaw	IPhCh	Ch	7
Institute of Metallurgy and Materials Science PASc (Instytut Metalurgii i Inżynierii Materiałowej PAN), Cracow (Kraków)	IMMI	T	6

* Polish names are given in parentheses

The basic bibliometric data, including the Hirsch index *h* and the tapered Hirsch index *h*_T_, for the Royal Society scientists are taken from Anderson et al. (2008). The data for Polish professors were collected by the present author and cover the period up to 2010. These basic data include the total number of papers *N*, research career length *t*, the number of citations *L* with self-citations and the Hirsch index *h*, but in the case of Polish professors the number of papers published in conferences/meetings and the number of papers published in limited-access journals such as those published in Polish, Russian or other regional languages are also given. The data for Lublin University of Technology (LUT) and non-Lublin University of Technology (non-LUT) professors, respectively, were collected in December 2010 whereas those for professors working in six traditional universities, eight technical universities and three research institutes of Polish Academy of Sciences (PASc), respectively, were collected during 10–15 March 2012 from Thomson Reuters’ ISI Web of Knowledge (Web of Science).

Anderson et al. (2008) chose the six Royal Society scientists from a consideration of their eminence in their scientific fields, whereas the present author selected the LUT and non-LUT professors arbitrarily taking into consideration solely their scientific, administrative or organizational activities. Among the non-LUT professors, four of them work in PASc, and two in Warsaw University of Technology (WUT). Three of the PASc professors also have university affiliations. Two of the nine professors selected from LUT have served before, and one is serving now, as prorectors for scientific affairs, three of them are deans of different faculties, while six have not been involved much in administrative work. One of the non-LUT professors has served before as a rector.

In contrast to the above arbitrarily chosen LUT and non-LUT professors, the selection of other Polish professors was made from the staff members of faculties/Departments of Physics, Chemistry and different technical sciences chosen from lists of ranking of traditional and technical universities for the year 2010, prepared jointly by Polish educational monthly “Perspektywy (Perspectives)” and daily “Rzeczpospolita (Republic)”. The universities were selected using the criterion: *y* = 3*x* − 2, where *x* is an integer ≥ 1. In the case of traditional universities, typically humanities universities were omitted from the selection. The three PASc institutes were selected according to their scientific fields (i.e., physics, chemistry, and metallurgy and materials engineering) from the list of PASc institutes prepared by the monthly “Forum Akademickie (Academic Forum)”.

Depending on whether the list of the members of the staff, available from the home page of a university or PASc institute, was prepared alphabetically or according to their academic positions, the professors, designated as *y*, were selected in two ways; (1) from the lists of the members of the staff of faculties/departments of selected universities and PASc institutes, using the criterion: *y* = 4*x* − 3 (where the integer *x* ≥ 1), and (2) in the case of small faculties, institutes and departments, their deans, directors and heads. In the former case, when the bibliometric data of a drawn professor could not be found in the Thomson Reuters’ database, his/her next neighbor was selected. In the case of UW, the selection of professors was mainly restricted to experimental solid state physics and physical chemistry. The publication output of professors active in agrophysics, biophysics and astrophysics was not taken into consideration due to their narrow domains. No special attention was made to trace the past or present administrative functions of all professors.

## Tapered index *h*_T_ and its relationship with Hirsch index *h*

### Ferrers diagrams and the tapered *h*_T_ index

**Fig. 1:**
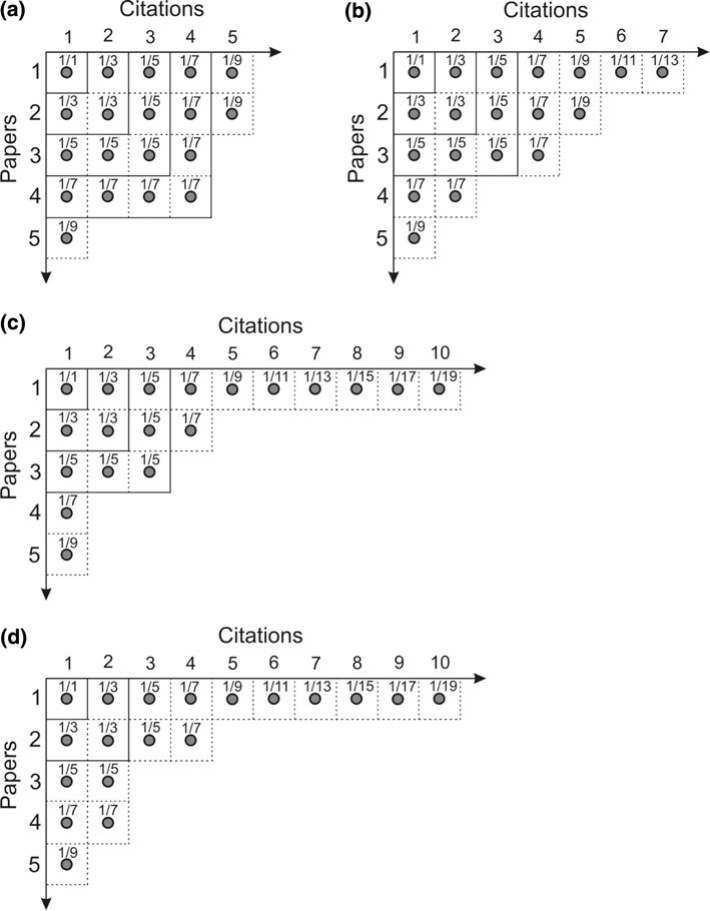
Examples of Ferrers diagrams representing citations of four fictitious scientists A, B, C and D, who published five papers each which earned a total of 19 citations. The order of decreasing citations of the papers is: **a** 5, 5, 4, 4, 1 for scientist A; **b** 7, 5, 4, 2 and 1 for scientist B; **c** 10, 4, 3, 1, 1 for scientist C; and **d** 10, 4, 2, 2, 1 for scientist D. Note that scientist A has *h* = 4, B and C have *h* = 3, whereas D has *h* = 2

The Hirsch index *h* corresponding to the citations of different papers published by an author may be illustrated conveniently by a Ferrers diagram which is a two-dimensional representation of citations *L* received by an author of *N* papers (for example, see Anderson et al. 2008; Franceschini and Maisano 2010b). In the Ferrers diagram the citations of different papers published by an author are represented as stacking of successive rows of two-dimensional squares of points (each point denotes a citation such that *l*_*n*_ is the number of citations of the *n*-th article in the ranking) starting at the top of the diagram from left to right followed successively downwards by papers with decreasing citations, and each row represents a partition of the *l*_*n*_ citations of the *n*-th paper. Four examples of Ferrers diagrams are presented in Fig. 1. The largest filled-in square of points in the Ferrers diagram is the Durfee square and has its side equal to the *h* index. The set of *h* most cited papers is also said to form the so-called *h*-core (Rousseau 2006).

As mentioned before, the main drawback of the *h* index is that it neglects all citations which fall outside the Durfee square (i.e., the *h*-core). These neglected citations are: (1) (*l*_*n*_ > *h*) citations, lying on the right of the Durfee square, received by (*n* < *h*) papers and (2) (*l*_*n*_ < *h*) citations, lying below the Durfee square, received by (*n* > *h*) papers. The citations falling in the two areas are also called areas of “big hits” and “sleeping beauties” and are designated here as BH and SB areas, respectively. Anderson et al. (2008) proposed a new version of the *h* index, called the tapered *h* index (*h*_T_) that accounts for citations lying outside the *h*-core. These authors also suggested a scoring mechanism to calculate *h*_T_ on equitable basis to that of *h*, thereby allowing direct comparison of the two measures of publication output.

Franceschini and Maisano (2010b) pointed out that the main drawbacks of the *h*_T_ index are: (1) its arbitrariness of the scoring system as the basis of its construction, which leads to its illusory discriminating power with respect to *h*, and (2) *h*_T_ contains no information on the shape of the corresponding Ferrers diagram. These authors proposed citation triad based on subdivision of the total number of citations *L* into three contributions: (1) *H* citations in the Durfee square, (2) *BH* citations to the right of the Durfee square (area BH) and (3) *SB* citations below the Durfee square (area SB). However, in view of arbitrariness in the scoring system for the citations of areas BH and SB, the discriminatory capability of these additional measures BH and SB in the citation triad can also be questioned. Consequently, as in the original version of the *h*_T_ index of Anderson et al. (2008), in this paper we shall follow the equitable system of scoring for citations in both BH and SB areas.

Figure 1 illustrates examples of the Ferrers diagrams of the citations of four fictitious scientists A, B, C and D, who published five papers each which earned them a total of 19 citations. Therefore, as far as the numbers of citations and papers of the four scientists are concerned, they are equally good. However, the citations of their papers are different, and, in the order of decreasing citations of the papers, are: 5, 5, 4, 4, 1 for scientist A; 7, 5, 4, 2, 1 for scientist B; 10, 4, 3, 1, 1 for scientist C; and 10, 4, 2, 2, 1 for scientist D. It may be seen that scientist A has *h* = 4, B and C have *h* = 3, whereas D has *h* = 2. Obviously, if *h* index is taken as a measure of the impact of papers of the above scientists, scientist A published the most influential papers, D the least influential ones, but B and C published papers which had similar impact and this impact was in between the impact of the papers of A and D. This is due to the fact that the citations which fall outside the Durfee square (i.e., the *h*-core) do not contribute to the *h* index.

It may be noted from Fig. 1 that increase in *h* − 1 index to *h* requires addition of (2*h* − 1) citations. For example, an increase in *h* index from one to two requires three citations whereas that of *h* from two to three requires five citations. Thus, the contribution of each citation in increasing *h* − 1 to *h* is 1/(2*h* − 1). Using this concept, Anderson et al. (2008) calculated the contributions of all citations in area BH (coming from papers with *l*_*n*_ > *h* for papers *n* < *h*) and area SB (originating from papers with *l*_*n*_ < *h* for papers *n* > *h*) to the *h*_T_ index. Obviously, *h*_T_ = *h* when there are no citations to the right of the Durfee square and below the Durfee square. However, *h*_T_ > *h* when there are citations in these areas.

### Relationship between *h* and *h*_T_ and the value of constant *A*

From Fig. 1 one finds that the value of *h*_T_ is 4.33, 4.215, 3.997 and 3.940 whereas that of *h* is 4, 3, 3 and 2 for scientists A, B, C and D, respectively. The corresponding values of the ratio *h*_T_/*h* are 1.083, 1.405, 1.332 and 1.970 such that the average *h*_T_/*h* = 1.448 ± 0.375, whereas those of the ratio *L*/*h* ^2^ = *A* are 1.188, 4.359, 3.539 and 4.75 for these scientists (average *L*/*h* ^2^ = 3.459 ± 1.596). Obviously, both *h*_T_/*h* and *A* have different values for different scientists and decrease in the sequence: A, C, B and D. We consider some more examples below. First we examine the data on *h*_T_, *h* and *L* reported by Anderson et al. (2008) and then those of LUT and non-LUT professors, respectively. The values of *h*_T_ in the latter case were calculated by the author following the procedure described above.

It was found that the values of the ratio *h*_T_/*h* for scientists of the Royal Society lies between 1.656 and 1.948. In view of relatively small differences in the value of *h*_T_/*h* for different authors, the values of *h* and *h*_T_ of the chosen scientists are related such that *h*_T_/*h* = 1.719 ± 0.115. In contrast to this, the ratio *h*_T_/*h* lies between 1.32 and 2.06 for LUT professors and between 1.59 and 1.99 for non-LUT professors, whereas the corresponding mean values of *h*/*h*_T_ are 1.698 ± 0.241 and 1.796 ± 0.152, respectively.

From the Ferrers diagrams presented in Fig. 1 it may be concluded that *h*_T_/*h* ratio increases with increasing *A*. Since determination of *h*_T_ is based on the total number of citations *L*, a similar trend in *h*_T_/*h* and *A* is indeed expected. However, in order to compare the value of the ratio *h*_T_/*h* for an author with his/her total number of citations *L*, one should take into account the ratio *L* ^1/2^/*h* = *A* ^1/2^ instead of *A*. Then, as discussed below, when various papers of an author receive citations “appropriately”, one anticipates the ratio *h*_T_/*h* comparable with that of *A* ^1/2^ (i.e., *h*_T_/*h* = *A* ^1/2^).

**Fig. 2:**
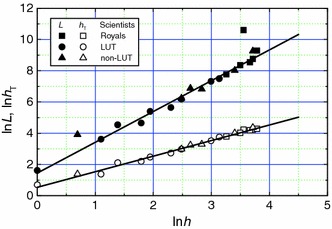
Best-fit plots of ln*L* and ln*h*_T_ against ln*h* for different scientists. Highly deviating data of *L* were omitted from the analysis

Figure 2 shows the relationship between constant *A* and *h*_T_/*h* ratio for Royal Society, and LUT and non-LUT scientists in the form of plots of ln*L* and ln*h*_T_ against ln*h*. It may be seen from Fig. 2 that ln*h*_T_ increases linearly with increasing ln*h* for these scientists. If the two highly deviating points are neglected, the data of ln*L* against ln*h* for different authors also follow a linear dependence. These linear dependencies may be described by the relation

2 $$ \ln X = k_{1} + k_{2} \ln h, $$

where *X* denotes cumulative citations *L* or tapered index *h*_T_, and *k*_1_ and *k*_2_ are, respectively, the intercept and the slope of the plots of ln*X* against ln*h*. It may be noted that Eq. (2) is indeed expected from the Hirsch relation (1) when *k*_1_ = ln*A* and *k*_2_ = 2. The best-fit constants for the linear plots according to relation (2) for the combined data for the above scientists are listed in Table 2. As seen from Table 2, the best-fit averaged values of *A* ^1/2^/*h* and *h*_T_/*h* obtained from the plots of Fig. 2 are 2.061 and 1.702, respectively, for the combined data for the above scientists. This implies that *A* ^1/2^ = 1.21*h*_T_ for the above scientists.

**Table 2:** Best-fit values of constants of relation (2)

Figure	Data	*k*_1_	e^*A*^	*k*_2_	*r* ^2^
2	ln*L*(ln*h*)	1.4459 ± 0.1534	4.246	1.9713 ± 0.0547	0.9935
	ln*h*_T_(ln*h*)	0.5318 ± 0.0692	1.702	0.9990 ± 0.0245	0.9949
3	ln*R*(ln*h*)	0.1506 ± 0.0772	1.1625	0.9856 ± 0.0275	0.9935
4	ln*L*(ln*h*)	1.2247 ± 0.1234	3.403	2.0081 ± 0.0502	0.9827
	ln*R*(ln*h*)	0.0400 ± 0.0617	1.041	1.0041 ± 0.0251	0.9827

It should be recalled here that addition of more citations from area BH or SB outside the Durfee square to the *h* index increases the value of *h*_T_. In other words, the tapered Hirsch index *h*_T_ does not distinguish between contributions from citations lying in areas BH and SB outside the Durfee square. This implies that the value of the *h*_T_ index with respect to the value of the original Hirsch index *h* is enhanced by a factor related to the constant *A* ^1/2^. However, in contrast to the *h* index which defines the largest filled-in square of points in the Ferrers diagram, both *h*_T_ index and constant *A* ^1/2^ provide no information on the shape of the corresponding Ferrers diagram (cf. Franceschini and Maisano 2010b). Consequently, the situation *A* ^1/2^ > *h*_T_/*h* results when contributions to the *h*_T_ index of an author come from citations lying in area BH or SB outside the Durfee square.

The examples considered above corroborate the observation in the Introduction that the value of the Hirsch constant *A* spans over a wide range. However, the situations with *A* = 1 and *A* > 1 occur when there are no citations and when there are citations, respectively, outside the Durfee square. The different values of *A* are due to different arrangements of citations outside the Durfee square.

## Concept of circular citation area and its radius *R*

Relation (1) between the total number of citations *L* of the papers of a scientist and his/her Hirsch index *h* implies that the citations form a close-packed arrangement of points or squares in two-dimensional citation space, as represented by Ferrers diagrams. The Durfee square or the *h*-core represents the area of the largest filled-in square of side *h*. The tapered index *h*_T_ is also based on the construction of Ferrers diagrams, in which contributions of citations outside the Durfee square to the total area are counted. However, if the contributions of citations outside the Durfee square are arranged as contributions around it to form a new square of sides *d* = *L* ^1/2^, *d* = *A* ^1/2^ *h*. Then *h*, *h*_T_ and *d*, all the three, are manifestation of two-dimensional square space, but the latter two measures include contributions of citations outside the Durfee square.

The approach of quantifying the scientific output of a scientist in terms of *h* index has been well recognized since it was proposed by Hirsch (2005). However, the main difficulty associated with it is that it is confined to the Durfee square in two-dimensional square space. Consequently, there are situations when an author has a low *h* index despite having published during his/her publication career a few outstanding paper(s) which received very high citations. In the case of Royal Society scientists (see Anderson et al. 2008), a typical example is ADB, who published 55 papers only which received *L* = 40,094 citations, but has a lower *h* = 28 in contrast to *h* = 31 of MREP, who published 89 papers which received *L* = 2,356 citations. In this specific example, the value of Hirsch constant *A* for ADB is more than nine times higher than that of MREP. Obviously, in this case, ADB has been penalized by the *h* factor despite the fact that his papers received 17 times more citations than MREP’s papers.

The above highly different values of *A* for citations of the publication output of different authors are a consequence of the definition of *h*. For example, in the case of the four virtual scientists of Fig. 1 with *L* = 19, the Hirsch constant *A* changes by a factor of 3.9 from 1.188 for scientist A to 4.359 for scientist B, but by a factor of 3.17 from 1.118 for scientist A to 3.539 for scientist C. Similarly, for *L* = 500 citations for a moderately active scientist with *h* = 12, *A* = 3.472, but different arrangements of citations of his/her papers in the Ferrers diagrams resulting in *h* = 11 and 13, *L* = 500 citations would give *A* = 4.132 and 2.959, respectively.

The above examples show that dispersion in the value of *A* is associated with the discrete nature of *h* index of the authors, as determined by the arrangements of the citations of their papers in the Ferrers diagrams. It may also be verified easily that dispersion in the value of *A* with changes in the discrete values of *h* by ± 1 decreases with increasing citations and increases with decreasing citations of scientists (see Fig. 4c). In order to overcome this drawback of *h* index, which is essentially associated with the square citation space of side *h*, we propose here a circular citation space such that the total citations *L* are now confined in a circle of radius *R*, described by

3 $$ L = \pi R^{2}, $$

where the constant π denotes a fixed value of *A*. For the sake of brevity, this circular citation area radius is referred to as the circular citation radius *R* hereafter.

Note that both *h*_T_ and *R* include all of the citations comprising citation areas H, BH and SB of an author, but *h*_T_ is also not an effective measure of the impact of his/her scientific output. Among the Royal Society authors for example (see Anderson et al. 2008), the *h*_T_ index of ADB (*h*_T_ = 68.18) is lower than that of RJJ (*h*_T_ = 72.03). This ranking is the same as that predicted by the *h* indexes of these two authors, although the papers of ADB received citations four times higher than those of RJJ. However, when *R* is taken as a measure of the impact, ADB is the unquestionable leader in the ranking of Royal Society scientists. In this sense, *R* is superior to *h*_T_. Moreover, *R* can be calculated immediately without constructing Ferrers diagram for the publication output of an author to calculate his/her *h*_T_ index.

From the citation data of different authors one may compare the values of citation radius *R* with Hirsch index *h* using Eqs. (1) and (2), i.e.,

4 $$ R = (A/\pi )^{ 1/ 2} h. $$

This relation between *R* and *h* predicts a linear dependence of ln*R* on ln*h* with slope *k*_2_ = 1 and intercept *k*_2_ = ln[(*A*/π)^1/2^]. The data of *R* against *h* for Royal Society scientists and LUT and non-LUT scientists were analyzed from the plot of ln*R* against ln*h*, omitting the highly deviating data of *R* and *L*. The best-fit values of constants *k*_1_ and *k*_2_ for the linear dependence are included in Table 2. The values of *k*_1_ ≈ 0 and *k*_2_ ≈ 1 imply that *R* ≈ *h.*

**Fig. 3:**
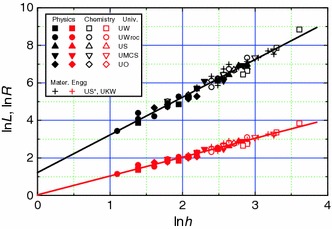
Best-fit plots of ln*L* (upper curve) and ln*R* (lower curve) against ln*h* for physics, chemistry and materials engineering professors working in traditional universities

The citation radius *R* is compared below with *h* index for different professors working in tradition universities, technical universities and PASc institutes considered in this paper. For the purpose of comparison we consider *L* and *R* as functions of *h*. Figure 3 shows a typical example of the linear dependencies of ln*L* and ln*R* on ln*h* for the data of traditional university professors of different specialities, where the plots are drawn with the best-fit values of constants *k*_1_ and *k*_2_ given in Table 2 for the entire 59 data points. Once again one finds that *R* ≈ *h*.

**Fig. 4:**
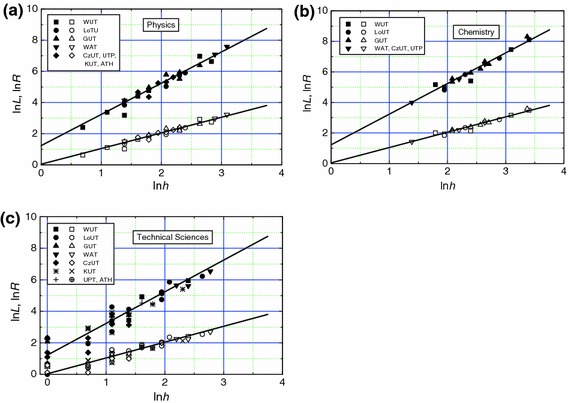
Dependence of ln*L* (upper curve) and ln*R* (lower curve) on ln*h* for **a** physics, **b** chemistry and **c** technical sciences professors working in technical universities. *Plots* are drawn with the best-fit values of parameters obtained for data of all scientists working in traditional universities

**Fig. 5:**
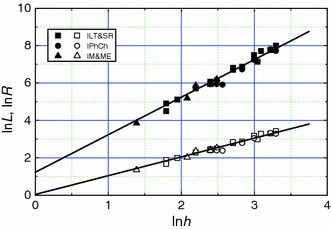
Dependence of ln*L* (upper curve) and ln*R* (lower curve) on ln*h* for physics, chemistry and technical sciences professors working in selected institutions of Polish Academy of Sciences. *Linear plots* are drawn with the best-fit values of parameters obtained for data of all professors working in traditional universities

Data of ln*L* and ln*R* on ln*h* for technical university and PASc professors are presented in Figs. 4a–c and 5, respectively. The plots are drawn with the best-fit values of the constants *k*_1_ and *k*_2_ given in Table 2 for traditional university professors. It may be seen that the data are well represented by the linear plots drawn with the values of *k*_1_ and *k*_2_ of Table 2 for physics and chemistry professors, but, corresponding to different values of *h*, the data are highly dispersed from the linear dependence in the case of technical sciences professors for ln*h* below about 2 (i.e., *h* below about 10). As mentioned above, these dispersions in ln*L* and ln*R* from the linear plots are associated with the discreteness of the *h* index.

We note that the same values of circular citation radius *R* and Hirsch index *h* for an author are expected when (*A*/*π*)^1/2^ = 1 (i.e., *A* = *π* = 3.142). This situation occurs in the case of citation data for a majority of scientists considered above. All deviations from the equality *R* = *h* result when (*A*/*π*)^1/2^ ≠ 1. When *A* ^1/2^ > 1.772, *R* > *h*, whereas when *A* ^1/2^ < 1.772, *R* < *h*. The citation data of real scientists and several fictitious scientists considered above can be explained in this way.

## Some general features of citations of publication output of Polish scientists

### Concepts of citation acceleration *a* and *R*-rate

It is well known that the cumulative citations *L*(*t*) and the Hirsch index *h*(*t*) of an author increase with his/her publication career *t*, the number *N* of published papers and their citability. Analysis of citation data of different authors using Hirsch relation (1), written in the form of Eq. (2), does not provide any useful information on the effects of the above factors. In order to account for the effects of such factors as publication time *t*, publication rate Δ*N* and scientific field, here we introduce and use concepts of citation acceleration *a* and circular citation radius rate (i.e., *R*-rate). For this purpose we follow the ideas of stochastic model of Burrell (2007a, b).

Using his stochastic model (Burrell 2007a, b) proposed that

5 $$ \frac{L(t)}{{t^{2} }} \approx \left( {\frac{\Updelta L}{2}} \right)\Updelta N, $$

and

6 $$ \frac{h(t)}{t} \approx (2\Updelta L)^{1/2}, $$

where Δ*N* is the average publication rate (papers published per year) and Δ*L* is the average citation rate (i.e., citations per year) of an author. According to these relations, the ratio *L*(*t*)/*t* ^2^, called here citation acceleration *a*, is proportional to Δ*N* with slope Δ*L*/2 whereas the ratio *h*(*t*)/*t*, called *h*-rate by Burrell (2007c), is a constant equal to (2Δ*L*)^1/2^. A relation similar to Eq. (6) may also be obtained from Eqs. (4) and (5) in the form

7 $$ \frac{R(t)}{t} \approx \left( {\frac{\Updelta N \cdot \Updelta L}{2\pi }} \right)^{1/2}, $$

implying that the ratio *R*(*t*)/*t*, called *R*-rate hereafter, for an author is also a constant equal to (Δ*N*·Δ*L*/2π)^1/2^.

It should be mentioned here that, recently, the present author (Sangwal 2012) used the concept of citation acceleration *a* to compare the publication output of different authors and interpret the meaning of an age-independent index proposed previously by Burrell (2007c), Kosmulski (2009) and Abt (2011) on the basis of average value of *h* index over time *t*. For the purpose of comparison, the author used the *a* ^1/2^ parameter which is essentially related to the circular citation radius *R*.

**Fig. 6:**
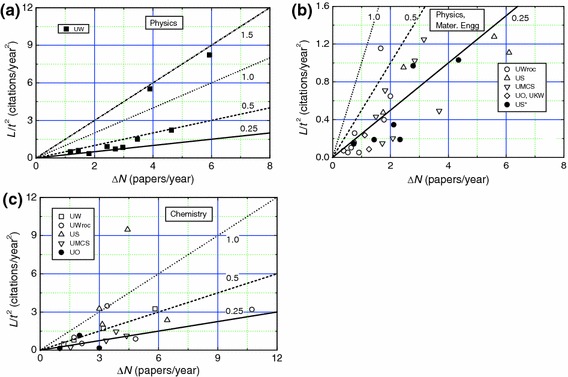
Dependence of *L*/*t* ^2^ on Δ*N* for **a**, **b** physics and materials engineering, and **c** chemistry professors working in traditional universities. *Slopes of plots* are indicated. In **b** materials engineering professors are indicated by *filled circles*

**Fig. 7:**
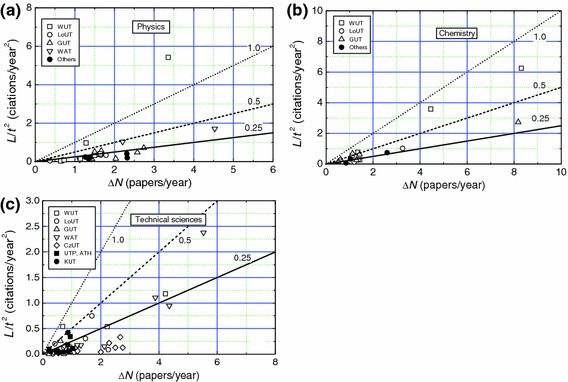
Dependence of *L*/*t* ^2^ on Δ*N* for **a** physics, **b** chemistry and **c** technical sciences professors working in technical universities. *Slopes of plots* are indicated

**Fig. 8:**
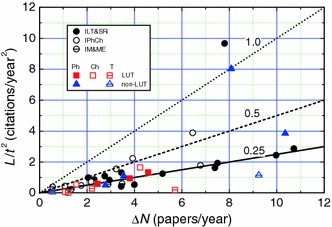
Dependence of *L*/*t* ^2^ on Δ*N* for physics, chemistry and technical sciences professors working in PASc institutes, LUT and non-LUT. *Slopes of plots* are indicated

Assuming that the above model applies in the case of the publication output of the authors considered in this work, we examined the trends of plots of *L*/*t* ^2^ against Δ*N* for different authors using Eq. (5). Figs. 6, 7 and 8 show the plots of *L*/*t* ^2^ against Δ*N* for professors of different specialities working in different traditional universities, technical universities and PASc institutes, respectively. The data of *L*/*t* ^2^ against Δ*N* for LUT and non-LUT professors are included in Fig. 8. The solid, dashed and dotted curves in the figures represent slopes of 0.25, 0.5 and 1, respectively.

From the above figures it may be seen that the citability of the papers, as determined by the slope Δ*L*/2 of the plots of *L*/*t* ^2^ against Δ*N* for various authors differs enormously with the upper limit of about 1.5 for physics and chemistry professors (see Figs. 6c and 7a), and about 0.5 for technical sciences professors (see Fig. 7c). However, the citability of a majority of the physics and chemistry professors lies between 0.2 and 0.5 (Figs. 6a–c, 7a, b and 8), whereas that of technical sciences professors is below 0.25 (Fig. 7c). These observations are consistent with the range of relatively high *h* values usually lying between 5 and 30 for physics and chemistry professors and relatively low *h* values lying frequently between 1 and 20 for technical sciences professors. Among the technical sciences professors, professors working in universities with low rankings in the hierarchy and specializing in electrical and high-power engineering have very low citability Δ*L*/2 and publication rate Δ*N* (see Fig. 7c).

Two interesting inferences follow from Figs. 6, 7, 8:

1. The values of Hirsch index *h* and circular citation area radius *R* of professors working in traditional and technical universities are comparable with those of professors working in research institutes of PASc. This feature is evident from the plots of Figs. 6, 7, 8, which reveal that physics and chemistry professors working in universities have comparable publication rate Δ*N* and citability Δ*L*/2 with those of their colleagues working in PASc research institutes, where they do not have teaching load. This observation is in conflict with the general belief that teaching load reduces the publication output. One of the possible explanations of this apparently contradictory observation lies in the fact that students contribute to research activities of professors in the universities whereas professors working in research institutes are devoid of such a contribution.
2. Physics and chemistry professors have a much higher *h* and *R* than their counterparts specialized in technical sciences. This inference also follows from the plots of Figs. 6, 7, 8, which show that the former professors have, in general, a higher publication rate Δ*N* and citability Δ*L*/2 than those of the latter professors. It was also found that, among the physics and chemistry professors, professors active in the subfields of nuclear physics, crystal chemistry or structural crystallography, and solution, environmental or food chemistry have relatively higher *h*, *R* and citability Δ*L*/2 than their colleagues, whereas, among the technical sciences professors, professors active in the subfields of electrical energy and high power engineering have very low *h*, *R* and citability Δ*L*/2 than their other colleagues. These observations are associated with the specifics of the trends of citations in these disciplines and subdisciplines.

### Influence of some other factors on citation acceleration *a*

Participation in conferences devoted to specific research areas is a part of the research activities of practically all scientists whereas, apart from involvement in research activities, bearing the administrative responsibility for some time by elected or appointed professors/scientists as rectors, deans and directors is a part of the functioning of departments, faculties and universities. In the former case, papers based on presentations in the conferences are usually published in special issues of journals and are included as the research output of an author. There are also researchers who, for different reasons, publish in non-English language journals which are not accessible to the common reader. Many of such limited-access journals are included in the databases. For example, among the Polish journals, Przegląd Elektrotechniczy (Electrical Engineering Review), Polimery (Polymers) and Rynek Energii (Energy Market) are typical examples of such journals which are included in the database available from Thomson Reuters’ ISI Web of knowledge (Web of Science) used in the present study. Therefore, it is of general interest to analyze the influence of conference papers, non-English papers and administrative functions of professors on the citability of their overall publication output. The concept of citation acceleration *a* = *L*/*t* ^2^ (and *R*-rate equal to (*a*/π)^1/2^; cf. Eq. (7)) is easy to understand and interpret scientometrically.

The role of above parameters in the publication output of the authors of different specializations can be analyzed from the plots of the citation acceleration *a* = *L*/*t* ^2^ as functions of fraction *f*_N_ of conference papers (defined as the number *N*_conf_ of papers published in special issues of journals divided by the total number *N* of papers), fraction *f*_NE_ of papers in limited-access journals (defined as the number *N*_NE_ of papers published in limited-access journals divided by the total number *N* of papers), and publication rate Δ*N* of scientists involved in administrative work. These dependencies are presented below.

**Fig. 9:**
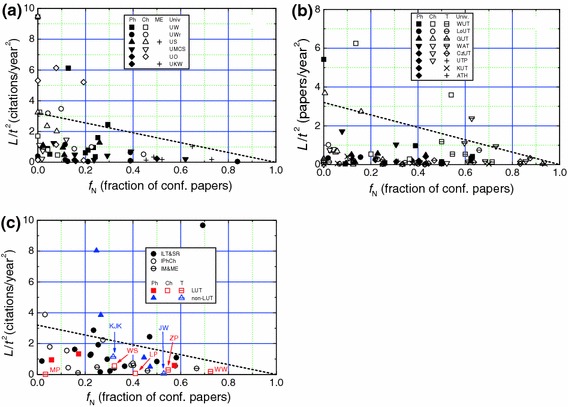
Dependence of *L*/*t* ^2^ on fraction *f*_*N*_ of conference papers for various scientists working in **a** traditional universities, **b** technical universities and **c** PASc institutes, LUT and non-LUT. *Dashed lines* denote slope −3.2. The value of *L*/*t* ^2^ = 3.2 at *f*_N_ = 0 corresponds to publication rate Δ*N* = 4 papers/year and citability 0.8. Total number of professors analyzed in the figures is 183

Figure 9a–c show the data of *L*/*t* ^2^ against the fraction *f*_N_ of conference papers for different groups of Polish professors. In the figures the dashed lines present a slope of −3.2. The initial value of *L*/*t* ^2^ = 3.2 at *f*_N_ = 0 was selected from a consideration of the reference value of *L*/*t* ^2^ = 1.5 for the nuclear physicist K. Pochucki of UW, who published all of his papers in regular issues. The selected value of *L*/*t* ^2^ = 3.2 corresponds to the average publication rate Δ*N* = 6.4 papers/year and moderate citability 0.5 (cf. Fig. 6a). However, as the citation data for physics and chemistry professors suggest, a more realistic value of *L*/*t* ^2^ is about 2 (see Fig. 9a, b), which corresponds to Δ*N* = 5 papers/year corresponding to the citability 0.4 encountered for average physics and chemistry professor (see Fig. 6). A decreasing trend of the citability of conference papers with increasing fraction *f*_*N*_ of conference is evident in these figures. Moreover, technical sciences professors have a higher tendency of publishing papers in proceedings of conferences than physics and chemistry professors (see Fig. 9b). The latter professors have comparable tendencies of publication of papers in conference issues of journals.

**Fig. 10:**
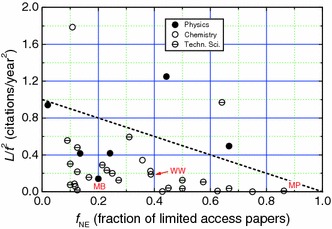
Dependence of *L*/*t* ^2^ on fraction *f*_NE_ of papers published by different professors in limited-access journals; *dashed line* denotes slope −1 where the value of *L*/*t* ^2^ = 1 at *f*_NE_ = 0 corresponds to Δ*N* = 2.5 papers/year and citability 0.4. Total number of professors analyzed is 42

The dependence of *L*/*t* ^2^ on fraction *f*_NE_ of papers published by different professors in limited-access journals is presented in Fig. 10. The dashed line denotes slope −1 where the value of *L*/*t* ^2^ = 1 at *f*_NE_ = 0 corresponds to a relatively low publication rate Δ*N* = 2.5 papers/year and citability 0.4. The data in Fig. 10 indicate that the citability of the papers show decreasing tendency with increasing *f*_NE_. The points indicated as MB, WW and MP denote the citation acceleration *a* of three professors, who have published major parts of their papers in limited-access journals; MB and MP in Russian-language journals whereas WW in Polish-language journals. These values of *a* are relatively low and are partly due the fact that these professors have been involved in administrative work as deans (see below).

**Fig. 11:**
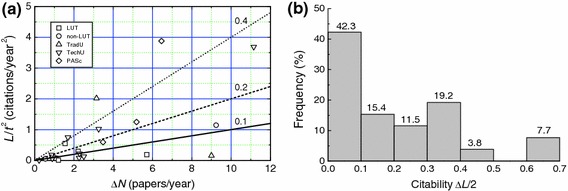
**a** Dependence of *L*/*t* ^2^ on Δ*N* for different professors involved in administrative work *linear plot* is drawn with the best-fit values of parameters obtained for data of all professors working in traditional universities. **b** Histograph of frequency of participation of Polish professors engaged in administrative work, with different citations. *Numbers* denote percentage of participation analyzed professors in different ranges of citability. Total number of professors analyzed is 26

Figure 11a shows the dependence of *L*/*t* ^2^ on Δ*N* for 26 professors involved in administrative work. The linear plots are drawn with the values of citability equal to 0.1, 0.2 and 0.4. The frequency of participation of professors in administrative work as a function of the citability of their papers is presented in Fig. 11b. It may be seen that the citability of about one-half of the professors in administrative work is less than 0.1 and is certainly very low. The publication rate Δ*N* of these professors is less than 2.4 papers/year (see Fig. 11b), which is relatively low.

From the above results, the following general features may be noted:

1. Increasing fraction of papers published by different authors in proceedings of conferences published as special issues of journals and in limited-access journals results in a lower citability of the papers (see Figs. 9 and 10).
2. Professors who have/had been engaged in administrative functions such as rectors, prorectors and deans, usually have *h* and *R* and relatively low values of publication rate Δ*N* and citability Δ*L*/2. This observation may be attributed to the fact that these professors devote a part of their time meant for research work to administration.

## Summary and conclusions

The nature of the Hirsch constant *A* in the classical relation *L* = *Ah* ^2^ between total number of citations *L* of the publication output of an author and his/her Hirsch index *h*, advanced by Hirsch (2005), is analyzed using data of the publication output and citations for six scientists elected to the membership of the Royal Society in 2006 and 199 selected physics, chemistry and technical sciences professors working in selected traditional universities, technical universities and PASc research institutes in Poland. It was found that different values of the Hirsch constant *A* for different scientists are associated with the discreteness of *h* and are related to the tapered Hirsch index *h*_T_ by *A* ^1/2^ ≈ 1.21*h*_T_. In order to overcome the drawback of a wide range of *A* associated with the discreteness of *h* for different authors, a simple index, the radius *R* of circular citation area, defined as *R* = (*L*/π)^1/2^ is proposed. It turns out that *R* ≈ *h*.

It is well known that the cumulative citations *L*(*t*) and the Hirsch index *h*(*t*) of an author increases with his/her publication career *t*, the number *N* of published papers and their citability. In order to account for the effects of such factors as publication time *t*, publication rate Δ*N* and scientific field, following the ideas of stochastic model of Burrell (2007a, b), the concept of citation acceleration *a* = *L*/*t* ^2^, related to the circular citation radius *R* by Eq. (7), is introduced and used to analyze the citation data of Polish professors. It was found that the citability, given by Δ*L*/2, lies between 0.2 and 0.5 and the publication rate Δ*N* < 4 for a majority of physics and chemistry professors (Figs. 6a–c, 7a, b and 8), whereas the citability Δ*L*/2 < 0.25 and Δ*N* < 2 for technical sciences professors (Fig. 7c). These observations are consistent with the range of relatively high values of *h* and circular citation radius *R* usually lying between 5 and 30 for physics and chemistry professors (Figs. 2, 3, 4a, b and 5) and relatively low values of *h* and circular citation radius *R* lying frequently between 1 and 20 for technical sciences professors (Figs. 3 and 4c). Among the technical sciences professors, professors working in the universities with low rankings in the hierarchy and specializing in electrical and high-power engineering have very low citability Δ*L*/2 and publication rate Δ*N* (see Fig. 7c. However, physics and chemistry professors working in universities have comparable publication rate Δ*N* and citability Δ*L*/2 with those of their colleagues working in PASc research institutes (see Figs. 6a–c, 7a, b and 8).

Analysis of the influence of conference papers, non-English papers and administrative functions of professors on the citability of their overall publication output revealed that increasing fraction of papers published by different authors in limited-access journals and in proceedings of conferences published as special issues of journals results in a decreasing citability of the papers (see Figs. 9 and 10). Similarly, professors who have/had been engaged in administrative functions such as rectors, prorectors and deans, usually have low *h* and *R* and relatively low values of publication rate Δ*N* and citability Δ*L*/2.

## Electronic supplementary material

Below is the link to the electronic supplementary material.

Supplementary material 1 (pdf 232 KB)
