# Supplementary material for: On the relationship between citations of publication output and Hirsch index h of authors: conceptualization of tapered Hirsch index hT, circular citation area radius R and citation acceleration a
Source: Scientometrics. 2012 Jul 10;93(3):987–1004. doi: 10.1007/s11192-012-0805-7 (PMC3495097; doi:10.1007/s11192-012-0805-7)
Supplement: Supplementary file 1 — Supplementary material 1 (pdf 232 KB) [file 11192_2012_805_MOESM1_ESM.pdf]

Appendixes

Appendix A1. Publication data and some citation indexes of selected Royal Society scientists

Scientist	*N* (years)	*L*	*h*	h_T	h_T/h	*A*	*R*
Badford D (DB)	78 (20)	6281	41	67.88	1.656	3.736	44.71
Becke AD (ADB)	55 (28)	40094	35	68.18	1.948	32.730	112.97
Lockwood M (ML)	176 (25)	5101	39	65.43	1.678	3.354	40.30
Jackson RJ (RJJ)	79 (36)	10778	44	72.03	1.637	5.567	58.57
Proctor MRE (MREP)	89 (31)	2356	26	44.32	1.705	3.485	27.38
Saibil HR (HRS)	80 (30)	4234	33	55.78	1.690	3.888	36.71

Appendix A2. Publication activity, citation parameters and circular citation area radius *R* of selected professors of Lublin University of Technology

Scientist	Function, Discipline	Papers *N* (in years)	*L*	*h*	h_T	h_T/h	*A*	*R*	Conf.
Gładyszewski G	Ph	54 (22)	281	10	15.30	1.530	2.810	9.458	31
Litak G	Ph	87 (19)	481	12	19.623	1.650	3.340	12.374	15
Sangwal K	Ph	152 (40)	1505	20	33.736	1.687	3.673	21.887	9 (+3)*
Kosmulski M	Ch	140 (33)	1790	23	42.419	1.844	3.384	23.870	–
Pawłowski L	PR, Dean, Ch	39 (36)	93	4	8.247	2.062	5.813	5.441	16 (+2)*
Stępniewski W	PR, Ch	31 (20)	220	7	11.806	1.687	4.490	8.368	10
Pater Z	PR, T	42 (19)	105	6	8.956	1.492	2.917	5.781	23 (+9)*
Pashechko M (MP)	Dean, T	29 (24)	5	1	2.01	2.01	5.00	1.262	1 (+25)*
Wójcik W (WW)	Dean, T (EE)	80 (14)	37	3	3.967	1.322	4.411	3.432	58 (+31)*

Appendix A3. Publication activity, citation parameters and circular citation area radius *R* of selected professors not affiliated to Lublin University of Technology

Scientist	Function, Discipline	Papers *N* (in years)	*L*	*h*	h_T	h_T/h	*A*	*R*	Conf.
Barnaś J	Ph	290 (28)	3017	30	55.001	1.833	3.352	30.989	77
Dietl T	Ph	292 (36)	10405	41	78.091	1.905	6.190	57.550	72
Krukowski S	Ph	103 (29)	916	17	27.025	1.590	3.170	17.075	46
Żytkiewicz Z	Ph	89 (32)	510	12	19.771	1.648	3.542	12.741	42
Kurzydłowski KJ	DTR, T	268 (29)	962	14	25.320	1.809	4.908	17.499	85
Woźnicki J (JW)	R, T	17 (31)	50	2	3.983	1.992	12.50	3.989	9

Appendix A4. Publication activity, citation parameters and circular citation area radius *R* of professors of selected traditional Polish universities

Institution	Author	Function, Discipl.	Papers *N* (in years)	*L*	*h*	*A*	*R*	Conf.
UW	Babiński A	Ph	57 (22)	320	9	3.951	10.09	12
	Baj M	Ph	58 (33)	353	12	2.451	10.60	14
	Kamińska M	Ph	111 (33)	1540	21	3.492	22.14	28
	Kowalczyk P	Ph	98 (34)	914	16	3.570	17.06	4
	Matulewicz T	DTR, Ph	55 (39)	851	13	5.036	16.46	11

	Napiórkowski M	DTR, Ph	40 (35)	573	11	4.734	13.51	2
	Pachucki K	Ph (N)	89 (16)	1851	26	2.738	24.27	0
	Satuła W	Ph (N)	78 (21)	2208	24	3.833	26.51	10
	Sosnowska I	Ph	90 (38)	1252	18	3.864	19.96	21
	Wysmolek A	Ph	102 (23)	1079	14	5.505	18.53	30
	Jackowska K	Ch	48 (41)	765	18	2.361	15.6	4
	Krygowski TM	Ch (SC)	268 (47)	6875	37	5.022	46.78	2
	Sadlej J	Ch (SC)	125 (40)	2593	27	3.557	28.73	0
	Skompska M	Ch	48 (29)	632	17	2.187	14.18	2
UWroc	Antczak G	Ph	16 (22)	113	6	3.139	6.0	0
	Chojcan J	Ph	18 (29)	81	4	5.063	5.08	7
	Gołek F	Ph	19 (37)	68	5	2.720	4.65	4
	Jędrzejewski J	DTR, Ph	26 (36)	194	7	3.959	7.86	3
	Koza Z	Ph	35 (22)	509	12	3.535	12.73	4
	Kucharczyk R	Ph	36 (19)	210	8	3.281	8.18	14
	Mozrzymas M	Ph	31 (22)	31	3	3.444	3.14	1
	Popowicz Z	Ph	64 (37)	516	13	3.053	12.82	0
	Stankiewicz B	Ph	31 (35)	59	4	3.688	4.33	26
	Filanowski A	Ch	51 (16)	782	15	3.476	15.78	5
	Grzeszczuk M	Ch	31 (19)	324	11	2.678	10.16	7
	Jakubas R	Ch	150 (32)	845	14	4.311	16.40	23
	Lis T	Ch (SC)	236 (23)	1544	18	4.765	22.17	10
	Orzechowski K	Ch	36 (18)	151	6	4.194	6.93	16
US	Kusz J	Ph	183 (31)	998	16	3.898	17.82	16
	Roleder K	DTR, Ph	76 (32)	916	18	2.827	17.08	8
	Talik E	Ph	162 (30)	1074	18	3.315	18.49	43
	Ujma Z	Ph	63 (37)	615	14	3.138	13.99	7
	Daszykowski M	Ch	31 (8)	464	12	3.222	12.15	0
	Krompiec S	Ch	24 (9)	208	8	3.250	8.14	0
	Machura B	Ch (SC)	103 (17)	604	13	3.574	13.87	4
	Polański J	DTR, Ch	63 (21)	808	15	3.591	16.04	5
	Bogdanowicz W	T	23 (17)	48	4	3.00	3.91	12
	Goryczka T	T	74 (18)	298	9	3.679	9.74	48
	Haneczok G	T	76 (37)	449	12	3.118	11.95	37
	Lelątko J	DTR, T	63 (28)	138	6	3.833	6.63	46
	Wokulska K	T	22 (31)	129	7	2.633	6.41	10
UMCS	Jalochowski M	Ph	69 (40)	1024	17	3.543	18.02	27
	Zuk J	Ph	50 (26)	116	6	3.222	6.08	31
	Budzyński M (MB)	Dean, Ph	55 (34)	152	6	4.222	6.96	13 (+11)*
	Baran A	Ph	48 (34)	441	10	4.410	11.85	14
	Murawski K	Ph	71 (27)	640	13	3.787	14.27	1
	Wysokiński KI	Ph	129 (37)	602	13	3.562	13.8	20
	Kamiński WA	R, Ph	60 (21)	451	13	2.669	11.98	4
	Chibowski E	Ch	150 (40)	2226	26	3.293	26.62	18
	Jańczuk B	Ch	162 (38)	1541	20	3.853	22.15	1
	Kozioł AE	Ch (SC)	97 (30)	629	13	3.722	14.15	2
	Matynia T	Ch	57 (38)	305	9	3.765	9.85	1 (+22)**
UO	Biedrzycki K	Ph	26 (37)	196	9	2.420	7.90	4
	Pietrzyk R	Ph	44 (36)	107	5	4.280	5.8	7
	Broda M	Ch	26 (38)	184	8	2.190	7.65	5
	Wojtaszek H	Ch	26 (21)	460	11	3.802	12.1	2
	Daszkiewicz	Ch	49 (37)	202	8	3.156	8.02	0
UKW	Fabisiak K	Ph	30 (28)	172	8	2.688	7.40	15
	Zienkiewicz M	T	39 (15)	190	8	2.969	7.78	1 (+25)**

* Papers in Russian.

** Papers in Polish.

Appendix A5. Publication activity, citation parameters and circular citation area radius *R* of professors of selected Polish universities of technology

Institution	Author	Function, Discipline	Papers *N* (in years)	*L*	*h*	*A*	*R*	Conf.
WUT	Bogusz W	Ph	36 (29)	761	17	2.633	15.56	14
	Gałązka-Friedman J	Ph	27 (38)	118	6	3.778	6.13	7
	Gniadek K	Ph	14 (22)	24	4	1.50	2.76	7
	Kosinski R	Ph	16 (15)	29	3	3.222	3.04	8
	Magierski P	Ph (N)	47 (15)	1063	14	5.423	18.39	0
	Petykiewicz J	Ph	11 (18)	11	2	2.750	1.87	1
	Woliński TR	Ph	24 (17)	82	5	3.280	5.11	15
	Wsiucionek M	Ph	46 (29)	322	10	3.220	10.12	29
	Brudzewski K	Ch (SC)	20 (19)	175	6	4.861	7.46	4
	Domańska U	Ch (OC)	191 (24)	3303	30	3.670	32.42	26
	Książczak A	Ch	33(24)	148	7	3.020	6.86	12
	Szatyłowicz H	Ch	23 (18)	223	11	1.843	8.43	1
	Wieczorek W	Ch (SC)	98 (23)	1737	22	3.589	23.51	53
	Olszyna A	T	40 (18)	174	7	3.551	7.44	24
	Swiątnicki W	T	11 (17)	138	5	5.520	6.63	6
	Trzaska M	T	14 (16)	32	4	2.00	3.19	10 (+2)*
	Wierzchoń T	T	76 (19)	382	11	3.157	11.03	38
LoUT	Bąk Z	Ph	12 (33)	46	4	2.875	3.83	6
	Kucharczyk W	Ph	59 (34)	367	11	3.033	10.81	7
	Prywer J	Ph	32 (21)	154	8	2.406	7.0	5
	Przanowski M	Ph	53 (33)	347	10	3.470	10.51	1
	Gębicki JL	Ch	48 (36)	338	10	3.380	10.37	11
	Jańkowski S	Ch	36 (31)	123	7	2.510	6.26	4
	Paneth P	Dean, Ch (OC)	101 (32)	981	18	3.028	17.67	2
	Bartoszewicz A	T	50 (35)	349	8	5.453	10.54	11 (+5)*
	Gawroński Z	T	8 (12)	15	3	1.667	2.19	1
	Hausman S	T	7 (21)	1	1	1.0	0.56	3 (+3)*
	Jantas R	T	43 (38)	186	7	3.796	7.69	1
	Korycki R	Dean, T	16 (18)	30	3	3.333	3.09	0
	Kucharski J	T	10 (18)	10	1	10.0	1.78	4 (+5)*
	Kulpiński P	T	8 (20)	72	3	8.00	4.79	1 (+2)*
	Mitura S	T	44 (27)	505	14	2.577	12.68	29
	Stempień Z	T	15 (15)	7	2	1.750	1.49	1 (+10)*
	Wendler B	T	29 (36)	115	7	2.347	6.05	10
	Wiak S	Dean, T	61 (28)	62	4	3.875	4.44	24 (+7)*
GUT	Perelomova A	Ph	30 (22)	60	4	3.750	4.37	4 (+20)*
	Rybicki J	Ph	67 (27)	326	8	5.094	10.19	29
	Sadowski W	Ph	61 (31)	142	6	3.944	6.72	38
	Sienkiewicz JE	Ph	35 (22)	249	10	2.490	8.90	8
	Szmytkowski R	Ph	63 (24)	377	10	3.770	10.95	2
	Zubek M	Ph	51 (35)	592	14	3.020	13.73 6	
	Chrzanowski W	Ch	18 (30)	256	8	4.00	9.03	0
	Darowcki K	Ch	139 (18)	789	14	4.026	15.85 22	
	Mazerski J	Ch	35 (32)	665	15	2.956	14.55 2	
	Namieśnik J	Dean, Ch (OC)	368 (34)	4014	29	4.773	35.74 2	
	Pawlak J	Ch	19 (37)	209	8	3.266	8.16	0
	Pilarczyk M	Ch	49 (39)	378	11	3.124	10.97 0	
	Stangret J	Ch	40 (29)	492	13	2.911	12.51 2	
	Chrzan PJ	T (EE)	10 (20)	27	3	3.00	2.93	5 (+1)*
	Imieliński K	T	8 (14)	43	4	2.688	3.70	2
	Jakubiuk K	Dean, T (EE)	8 (36)	8	1	8.00	1.596	1 (+5)*
	Nieznański J	T (HPE)	23 (28)	41	3	4.556	3.61	10
	Serbeński W	T	14 (16)	10	1	10.00	1.77	13
	Swędrowski L	T (EE)	3 (15)	0	0	0	0	1
	Świsulski D	T (EE)	8 (12)	0	0	0	0	2 (+6)*

WAT	Demianiuk M	Ph	75 (35)	1188	18	3.667	19.45	23
	Kłosowicz S	Ph	27 (25)	62	4	3.875	4.44	11
	Rogalski A	Ph	154 (35)	1976	22	4.083	25.08	12
	Bojar Z	Ch	23 (28)	54	4	3.375	4.15	12
	Bielecki Z	T	36 (18)	43	3	4.778	3.70	22
	Ciosek J	T	21 (17)	44	3	4.889	3.74	18
	Fiedorowicz H	T	94 (18)	687	16	2.684	14.79	59
	Madura H	T	19 (17)	45	4	2.813	3.78	13
	Mierczyk Z	T	74 (18)	273	11	2.256	9.32	54
	Zendzian W	T	62 (17)	283	9	3.494	9.49	37
CzUT	Pawlik P	Ph	37 (17)	106	5	4.240	5.81	16 (+5)*
	Zbroszczyk J	Ph	72 (32)	189	7	3.857	7.76	29
	Bala H	Ch	78 (31)	670	14	3.418	14.60	2
	Borowik L	T (EE)	2 (2)	0	0	0	0	0 (+2)*
	Dyja H	Dean, T	64 (29)	170	7	3.469	7.36	30 (+8)*
	Duś-Sitek M	T	7 (14)	3	1	3.00	0.98	3
	Frączek T	T	6 (17)	10	2	2.50	1.78	5
	Minkina W	T (EE)	9 (21)	23	4	1.438	2.71	4
	Muskalski Z	T	8 (4)	3	1	3.00	0.98	7 (+1)*
	Nitkiewicz Z	T	24 (21)	24	3	2.667	2.76	12
	Popławski T	T (EE)	11 (5)	2	1	2.00	0.80	7 (+3)*
	Rusek A	Dean, T (EE)	10 (5)	2	1	2.00	0.80	5 (+5)*
	Sawicki A	T (HPE)	6 (12)	4	2	1.00	1.13	2
	Stradomski Z	T	6 (9)	10	1	10.00	1.78	5 (+1)*
	Szkutnik J	T (HPE)	20 (11)	4	1	4.00	1.13	10 (+9)*
UTP	Czajkowski G	Ph	43 (35)	277	9	3.420	9.39	16
	Siuda R	Ph	39 (30)	130	6	3.611	6.43	12
	Ławrynowicz Z	T	2 (10)	8	2	2.00	1.60	-
	Szymura J	Ch	1 (36)	0	0	0	0	-
	Wrzyczczyński A	Ch	28 (28)	250	9	3.086	8.92	3 (+10)*
KUT	Pastusiak W	Ph	21 (31)	78	6	2.167	4.98	2
	Hryniewicz T	Dean, Ch	34 (39)	85	6	2.361	5.20	2 (+4)*
	Sienicki W	Ch	9 (34)	51	3	5.667	4.03	0 (+1)*
	Borkowski J	T	4 (3)	0	0	0	0	0 (+3)*
	Gulbiński W	T	20 (24)	222	10	2.220	8.41	2 (+4)*
	Kukiełka L	T	16 (23)	19	2	4.750	2.46	15
	Ratajski J	T	8 (18)	18	2	4.50	2.39	3
	Wilczyński B	T	10 (19)	14	3	1.556	2.11	7
ATH	Rabiej S	Ph	41 (30)	203	8	3.172	8.04	9
	Broda J	DTR, T	19 (23)	88	6	2.444	5.29	6
	Janicki J	T	30 (30)	92	5	3.680	5.41	13
	Pielesz A	T	18 (20)	124	5	4.960	6.28	4

* Papers in regional journals including Russian-language journals.

Appendix A6. Publication activity, citation parameters and circular citation area radius *R* of professors of selected institutes of Polish Academy of Sciences

Institution	Author	Function, Discipline	Papers *N* (in years)	*L*	*h*	*A*	*R*	Conf.
ILTSR	Baran J	Ph (SC)	255 (35)	2227	22	4.601	26.62	65
	Cichorek T	Ph	56 (20)	302	9	3.728	9.80	28
	Ciszek M	Ph	38 (33)	166	7	3.388	7.27	10
	Dereń PJ	Ph	55 (21)	436	11	3.603	11.78	32

	Hreniak D	Ph	78 (11)	967	17	3.346	17.54	54
	Jeżowski A	DTR, Ph	140 (28)	911	15	4.049	17.03	31 (+62)*
	Kaczorowski D	Ph	268 (26)	1792	20	4.480	23.88	63
	Klamut PW	Ph	41 (21)	395	11	3.264	11.21	12
	Pietraszko A	Ph (SC)	281 (39)	2345	25	3.752	27.32	44
	Strek W	Ph	349 (36)	2992	27	4.104	30.86	164
	Suski W	Ph	156 (40)	817	15	3.631	16.13	57
	Wiśniewski P	Ph	23 (21)	90	6	2.50	5.35	7
	Zaleski A	Ph	62 (19)	135	6	3.750	6.556	20 (+15)*
	Grabowska H	Ch	53 (24)	460	12	3.194	12.10	1 (+2)*
	Kępiński L	Ch	107 (32)	1257	21	2.850	20.00	24
IPhCh	Duś R	Ch	84 (38)	840	17	2.907	16.35	33
	Hołyst R	DTR, Ch (SC)	155 (25)	2239	27	3.071	26.70	5
	Janik-Czachor M	Ch	51 (14)	378	11	3.124	10.97	14
	Lipkowski J	Ch (SC)	251 (38)	2445	25	3.912	27.90	13 (+27)*
	Nowakowski R	Ch	50 (24)	382	12	2.653	11.03	20
	Opałło M	Ch	97 (31)	1392	20	3.480	21.05	12
	Zakroczymski T	Ch	31 (30)	369)	13	2.183	10.84	4
IMMI	Bełtowska E	T	6 (12)	47	4	2.938	3.87	4 (+2)*
	Gąsior W	T	56 (33)	491	12	3.410	12.50	14 (+7)*
	Major B	T	52 (40)	355	9	4.383	10.63	24 (+12)*
	Morgieł J	T	78 (28)	405	11	3.347	11.335	45 (+7)*
	Pawłowski A	T	47 (42)	178	8	2.781	7.53	8 (27)*
	Zięba P	DTR, T	87 (26)	371	11	3.066	10.87	35 (+27)*

* Papers in regional journals.
